# Contribution of birth weight to mental health, cognitive, and socioeconomic outcomes: a two-sample Mendelian randomisation

**DOI:** 10.1192/bjo.2021.169

**Published:** 2021-06-18

**Authors:** Massimiliano Orri, Jean-Baptiste Pingault, Gustavo Turecki, Anne-Monique Nuyt, Richard E Tremblay, Sylvana M Côté, Marie-Claude Geoffroy

**Affiliations:** 1 Bordeaux Population Health Research Centre, Mcgill University; 2 University College London; 3 Mcgill University; 4 University of Montreal

## Abstract

**Aims:**

Low birth weight is associated with adult mental health, cognitive, and socioeconomic problems. However, the causal nature of these associations remains difficult to establish due to confounding. We aimed to estimate the contribution of birth weight to adult mental health, cognitive, and socioeconomic outcomes using two-sample Mendelian randomisation, an instrumental variable approach strengthening causal inference.

**Method:**

We used 48 independent single-nucleotide polymorphisms as genetic instruments for birth weight (N of the genome-wide association study, 264 498), and considered mental health (attention-deficit hyperactivity disorder [ADHD], autism spectrum disorders, bipolar disorder, major depressive disorders, obsessive-compulsive disorder, post-traumatic stress disorder [PTSD], schizophrenia, suicide attempt), cognitive (intelligence), and socioeconomic (educational attainment, income, social deprivation) outcomes. We performed a two-sample Mendelian randomisation using the random-effect Inverse Variance Weighing method as primary analysis, supplemented by a wide range of sensitivity analyses, including Egger regression, weighted median, and Pleiotropy Residual Sum and Outlier. Results were considered statistically significant after accounting for multiple testing using False Discovery Rate (q = 0.05).

**Result:**

After correction for multiple testing, we found evidence for a contribution of birth weight to ADHD (OR for 1 SD-unit decrease [~464 grams] in birth weight, 1.29; CI, 1.03–1.62), PTSD (OR = 1.69; CI = 1.06–2.71), and suicide attempt (OR = 1.39; CI = 1.05–1.84), as well as for intelligence (β= –0.07; CI= –0.13; –0.02), and socioeconomic outcomes, ie, educational attainment (β=−0.05; CI= –0.09; –0.01), income (β=−0.08; CI= –0.15; –0.02), and social deprivation (β=0.08; CI = 0.03; 0.13). However, no evidence was found for a contribution of birth weight to the other examined mental health outcomes. Results were consistent across main and sensitivity analyses.

**Conclusion:**

These findings support that birthweight could be an important element on the causal pathway to mental health, cognitive and socioeconomic outcomes. Early interventions targeting birth weight may therefore have a positive impact on promoting mental health and improving socioeconomic outcomes.

This project has received funding from the European Union's Horizon 2020 research and innovation programme under the Marie Skłodowska-Curie grant agreement No 793396

